# Efeito Agudo do Exercício Intervalado *versus* Contínuo sobre a Pressão Arterial: Revisão Sistemática e Metanálise

**DOI:** 10.36660/abc.20190107

**Published:** 2020-07-28

**Authors:** Raphael José Perrier-Melo, Eduardo Caldas Costa, Breno Quintella Farah, Manoel da Cunha Costa

**Affiliations:** 1 Faculdade Pernambucana de Saúde RecifePE Brasil Faculdade Pernambucana de Saúde, Recife, PE - Brasil; 2 Universidade Federal do Rio Grande do Norte NatalRN Brasil Universidade Federal do Rio Grande do Norte - Educação Física, Natal, RN - Brasil; 3 Universidade Federal Rural de Pernambuco RecifePE Brasil Universidade Federal Rural de Pernambuco, Recife, PE - Brasil; 4 Universidade de Pernambuco RecifePE Brasil Universidade de Pernambuco - Educação Física, Recife, PE – Brasil

**Keywords:** Hipertensão, Pressão Arterial, Hipotensão Pós Exercício, Terapia por Exercício, Exercício, Revisão

## Abstract

**Fundamento:**

O exercício aeróbio contínuo (EC) é uma das principais recomendações não farmacológicas para prevenção e tratamento da hipertensão arterial sistêmica. O EC é seguro e eficaz para reduzir a pressão arterial cronicamente, assim como nas primeiras horas após sua realização, fenômeno conhecido por hipotensão pós-exercício (HPE). O exercício intervalado (EI) também gera HPE.

**Objetivo:**

Essa revisão sistemática e metanálise buscou comparar a magnitude da HPE entre o EC e EI em adultos.

**Métodos:**

Realizou-se uma revisão sistemática de estudos publicados em revistas indexadas nas bases *PubMed, Web of Knowledge, Scopus* e CENTRAL até março de 2020 que compararam a magnitude da HPE entre o EC *versus* EI. Foi definida HPE entre 45 e 60 minutos pós-exercício. As diferenças entre grupos sobre a pressão arterial foram analisadas por meio do modelo de efeito aleatório. Os dados foram reportados como diferença média ponderada (WMD) e 95% de intervalo de confiança (IC). Valor *p* menor que 0,05 foi considerado estatisticamente significativo. A escala TESTEX (0 a 15) foi usada para verificação da qualidade metodológica dos estudos.

**Resultados:**

O EI apresentou HPE de maior magnitude sobre a pressão arterial sistólica (WMD: -2,93 mmHg [IC95%: -4,96, -0,90], *p* = 0,005, I^2^ = 50%) e pressão arterial diastólica (WMD: -1,73 mmHg [IC95%: -2,94, -0,51], *p* = 0,005, I^2^ = 0%) quando comparado ao EC (12 estudos; 196 participantes). A pontuação dos estudos na escala TEXTEX variou entre 10 e 11 pontos.

**Conclusões:**

O EI gerou HPE de maior magnitude quando comparado ao EC entre 45 e 60 minutos pós-exercício. A ausência de dados sobre eventos adversos durante o EI e EC nos estudos impede comparações sobre a segurança dessas estratégias. (Arq Bras Cardiol. 2020; 115(1):5-14)

## Introdução

A hipertensão arterial sistêmica (HAS) acomete 30 a 40% da população mundial.^1 , 2^ No Brasil, sua prevalência varia de 22,3 a 43,9%, atingindo mais de 60% dos idosos.^3 , 4^ A HAS está diretamente associada à incidência de doenças cardíacas e cerebrovasculares,^3^ responsáveis por aproximadamente 20% das mortes em indivíduos acima de 30 anos,^5^ além de gerar um custo de 30,8 bilhões de reais por ano.^6^ Modificações no estilo de vida, incluindo exercício físico, alimentação saudável, redução do peso corporal e cessação do tabagismo têm sido fortemente recomendadas para prevenção e tratamento da HAS.^1 , 3^ De fato, modificações no estilo de vida geram reduções nos níveis de pressão arterial (PA), o que reduz o risco de eventos cardiovasculares.^3 , 7 , 8^

Em relação ao exercício físico, as diretrizes para prevenção e tratamento da HAS recomendam exercícios aeróbios realizados de forma contínua (EC), principalmente de intensidade moderada, por serem seguros e eficazes para redução dos níveis de PA, melhora do perfil de risco cardiovascular e metabólico, além de aumentar a aptidão cardiorrespiratória.^3 , 9^ Os efeitos anti-hipertensivos do EC podem ocorrer de forma aguda,^10 , 11^ fenômeno conhecido como hipotensão pós-exercício (HPE), e de forma crônica, após a realização de diversas sessões de exercício físico ao longo de semanas ou meses.^12 , 13^ Nos últimos anos, tem sido dada atenção especial aos exercícios que podem potencializar a magnitude e duração da HPE, tendo em vista que esse efeito pode gerar redução da sobrecarga cardiovascular nas horas subsequentes à sessão de exercício, o que pode reduzir o risco de eventos cardiovasculares.^14 , 15^ Além disso, estudos mais recentes têm demonstrado que indivíduos que apresentam HPE de maior magnitude após uma sessão de exercício tendem a apresentar maior redução da PA em repouso após semanas de treinamento (ou seja, maior efeito crônico).^16^ Portanto, a magnitude da HPE parece predizer a magnitude do efeito anti-hipertensivo crônico, o que representa importante aplicabilidade prática.

A HPE pode ocorrer com diferentes “doses” de exercício físico, tanto aeróbios quanto de força.^16^ Em relação aos exercícios aeróbios, uma revisão sistemática e metanálise anterior^11^ demonstrou que a HPE ocorre após a realização de EC e EI, apesar de ser principalmente documentada após EC, que é a base das recomendações para prevenção e tratamento de HAS.^3 , 9^ Entretanto, nos últimos anos o EI, seja em intensidade vigorosa ou máxima (“ *all out* ”), tem sido considerado uma alternativa ao EC para melhora de diversos parâmetros cardiovasculares, tais como capacidade cardiorrespiratória,^17^ função vascular^18^ e PA clínica.^19^ Porém, é importante destacar que não foram realizadas comparações diretas sobre os efeitos agudos do EC e do EI sobre a PA. Logo, não está claro se há superioridade do efeito anti-hipertensivo agudo entre os exercícios, o que constitui uma importante lacuna de conhecimento, uma vez que pode auxiliar profissionais tanto na prevenção quanto no tratamento da HAS. Portanto, o objetivo dessa revisão sistemática e metanálise foi comparar a magnitude da HPE entre EC e EI em adultos.

## Métodos

### Estratégia de busca na literatura

A revisão sistemática foi realizada seguindo as diretrizes do *Preferred Reporting Items for Systematic Reviews and Meta-analysis* (PRISMA).^20^ A busca dos artigos foi realizada nas bases eletrônicas *PubMed, Web of Knowledge, Scopus* e CENTRAL. A estratégia de busca utilizou os seguintes descritores e termos livres: *“high intensity interval training” [MeSH Terms] OR “high intensity interval exercise” [TIAB] OR “aerobic interval training” [TIAB] OR “aerobic interval exercise” [TIAB] OR “sprint training” [TIAB] OR “sprint” [TIAB] OR “sprint exercise”[TIAB] OR “sprint interval exercise” [TIAB] AND “blood pressure” [MeSH Terms] OR “post-exercise hypotension” [Mesh Terms] OR “postexercise hypotension” [Mesh Terms] OR “hypotension” [Mesh Terms]* . Todos os processos de busca, seleção e avaliação dos artigos foram feitos de forma duplicada e independente.

### Critério de elegibilidade

Os critérios de elegibilidade foram estabelecidos de acordo com a questão PICOS ( *Population, Intervention, Comparator, Outcomes e Study Design* ).

### População – Population

Essa revisão incluiu estudos que envolveram adultos (18 anos ou mais) de ambos os sexos, sem restrição quanto ao nível de atividade física e classificação da PA (normotensos, pré-hipertensos e hipertensos). Os valores médios de PA sistólica e diastólica pré-exercício foram utilizados para classificação dos indivíduos quanto à PA, seguindo-se os mesmos procedimentos de outras revisões sistemáticas^19 , 21^ e da 7^a^ edição das Diretrizes Brasileiras de Hipertensão.^3^

### Intervenção – Intervention

O esquema de classificação para EI proposto por Weston et al.^22^ foi utilizado para definição dos critérios de elegibilidade para essa intervenção. De acordo com essa proposição, repetidos estímulos em intensidade vigorosa (80 a 100% da frequência cardíaca de pico - FCpico) intercalados com períodos de recuperação (ativa ou passiva) são classificados como exercício intervalado de alta intensidade ( *high-intensity interval training* ), e estímulos máximos ( *“all out”;* ou acima da carga do consumo de oxigênio de pico -VO_2_pico) intercalados com períodos de recuperação (ativa ou passiva) são classificados como exercício intervalado de *sprint (sprint interval exercise* ). Estudos que utilizaram o percentual do VO_2_pico, VO_2_ de reserva ou percepção subjetiva de esforço (PSE) equivalentes a 80 a 100% da FCpico, de acordo com o Colégio Americano de Medicina do Esporte,^23^ foram considerados elegíveis, assim como os protocolos “ *all out* ”. Estudos que apresentaram intervenções associadas ao EI como outra forma de exercício (p. ex., exercício de força) ou estratégia nutricional não foram considerados para inclusão.

### Comparador – Comparator

O EC foi considerado comparador do EI. Estudos que utilizaram o percentual do VO_2_pico, VO_2_ de reserva ou PSE equivalentes a intensidade moderada (ou seja, 64 a 76% da FCpico) ou intensidade vigorosa (77 a 95% da FCpico) foram considerados elegíveis. Estudos que apresentaram intervenções associadas a EC, como outra forma de exercício ou estratégia nutricional, não foram considerados para inclusão.

### Desfechos

O desfecho primário dessa revisão foi a PA clínica, aferida entre 45 e 60 minutos após o exercício. Esse tempo pós-exercício foi definido considerando-se que a maioria dos estudos que investigaram os efeitos do EC e do EI incluiu medidas dentro desse período. Portanto, mesmo que o estudo tenha analisado a PA além de 60 minutos pós-exercício, essa medida não foi considerada para metanálise.

### Desenho do Estudo

Foram considerados estudos cruzados, envolvendo uma sessão de EC e EI, ordem de realização randomizada, em língua inglesa ou portuguesa. A busca foi realizada sem limite de data e foi encerrada em março de 2020.

### Extração de dados

Para extração dos dados dos artigos incluídos, foi utilizada uma planilha eletrônica, de acordo com os critérios de elegibilidade, de forma duplicada e independente. Em caso de discordância, convocava-se reunião e era estabelecido consenso entre os pesquisadores. As características dos participantes do estudo (idade, sexo, índice de massa corporal, nível de atividade física, classificação da PA), as características das sessões de exercício (modalidade, ambientes, duração, intensidade e tempo despendido na sessão de treino), o método de aferição da PA e o período de aferição da PA pós-exercício foram extraídos e registrados. Dados ausentes nos textos foram solicitados diretamente aos autores.

### Avaliação da qualidade metodológica dos estudos

A escala *Tool for the assEssment of Study qualiTy and reporting in Exercise* (TESTEX) foi utilizada para avaliação da qualidade metodológica dos estudos incluídos,^24^ também de forma duplicada e independente. Em caso de discordância, fazia-se reunião e era estabelecido consenso entre os pesquisadores.

### Síntese quantitativa

**Figura 3:**
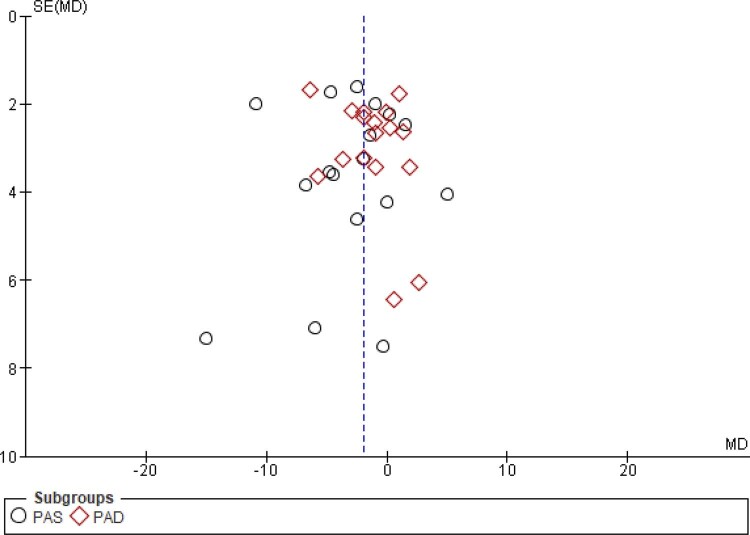
Funnel plot da comparação do exercício intervalado (EI) versus exercício contínuo (EC) sobre a pressão arterial (PA).

As mudanças [pós (-) pré-intervenção] da PA clínica foram extraídas de cada estudo e expressas em média ± desvio padrão. Os dados foram reportados como diferença média ponderada ( *weighted mean difference;* WMD) e intervalo de confiança (IC) de 95%. A heterogeneidade (I^2^) entre os estudos foi calculada. Valores acima de 75% e *p* < 0.10 foram utilizados para indicar alta heterogeneidade.^25^ O modelo de efeito aleatório (random-effect) foi adotado na presença de baixa ou alta heterogeneidade. Viés de publicação foi avaliado por meio do gráfico de funil ( Figura 3 ). Para realização da metanálise, foi utilizado o software *Review Manager* ( *RevMan* 5.3, *Nordic Cochrane* , Dinamarca). Dois estudos não reportaram os valores de desvio-padrão nos momentos pré- e pós-intervenção.^26 , 27^ Nesse caso, os valores foram estimados a partir das recomendações de Follman et al.^28^ Para tal, foi adotado como base o estudo de Costa et al.^29^ Em todas as análises, o nível de significância adotado foi de 5%.

## Resultados

### Estudos incluídos

**Figura 1:**
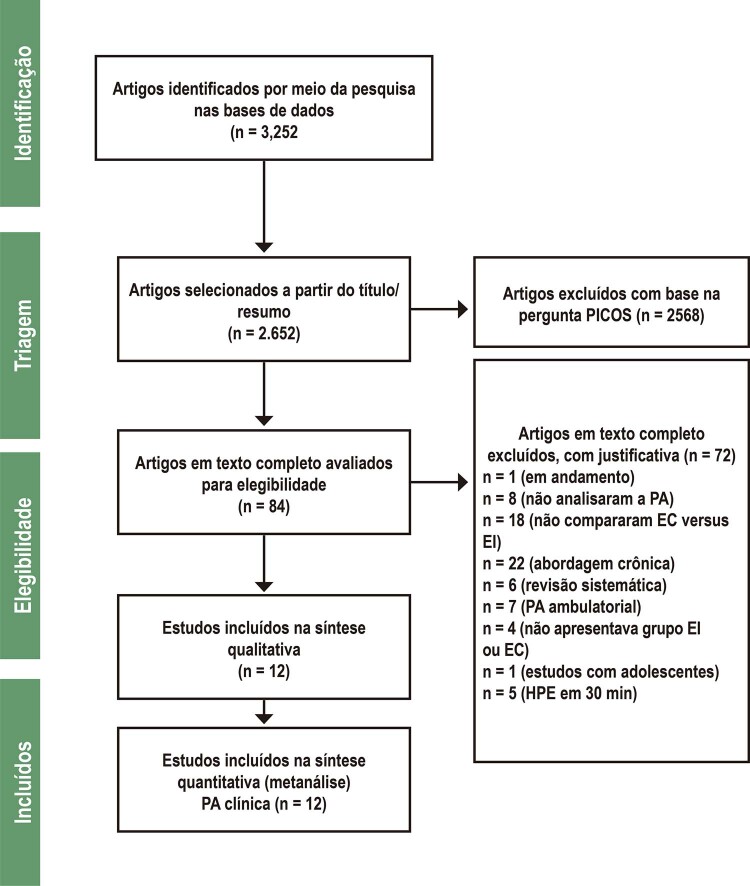
Fluxograma PRISMA dos estudos selecionados. PA: pressão arterial; EC: exercício aeróbio contínuo; EI: exercício intervalado; HPE: hipotensão pós-exercício.

A estratégia de busca identificou 3,252 artigos para análise inicial. Após a triagem dos títulos, resumos e exclusão dos resultados duplicados, foram selecionados 84 estudos para análise completa do texto. Desses, 72 não atenderam os critérios de elegibilidade para inclusão no estudo. Adicionalmente, um estudo não publicado foi incluído nas análises.^30^ A Figura 1 apresenta o fluxograma dos resultados da pesquisa.

### Características dos participantes

Os 12 artigos incluídos analisaram a PA clínica como desfecho principal e nenhum deles reportou efeitos adversos (n = 196; idade entre 20 e 75 anos; IMC entre 21,2 e 33,0 kg/m^2^ ).^26 , 27 , 29^ Desses, três estudos envolveram 46 normotensos (n = 23 mulheres),^26 , 29 , 34^ com idade média de 32,67 anos e IMC médio de 24,52 km/m^2^. A PA sistólica e diastólica média em repouso foi de 118/65,46 mmHg no EI e de 117,27/64,73 mmHg no EC. Seis estudos envolveram 89 pré-hipertensos (n = 1 mulher),^27 , 31 - 33 , 36 , 37^ idade média de 29,15 anos, IMC médio de 24,68 km/m^2^. A PA sistólica e diastólica média em repouso foi de 127,22/73,12 mmHg no EI e 126,72/73,22 mmHg no EC. Quatro estudos envolveram 61 hipertensos (n = 34 mulheres),^30 , 34 , 35 , 38^ idade média de 60,67 anos, IMC médio de 29,97 km/m^2^, e todos faziam uso de medicação anti-hipertensiva.

Em relação à aferição da PA, dos 12 estudos incluídos, quatro utilizaram o método auscultatório (~33%), enquanto os demais estudos utilizaram o método oscilométrico por equipamento automático. Todos os estudos utilizaram estatística inferencial, adotando valor de *p* ≤ 0,05. Na Tabela 1 e 2 estão as informações adicionais sobre as características dos estudos e das intervenções.

**Tabela 1:** Características dos participantes dos estudos incluídos

Autores	Participantes	Homens (%) / Mulheres (%)	Idade (anos)	IMC (kg/m^2^)	Característica da amostra
Pimenta et al.^38^	n=20 (15 mulheres)	25%/75%	51±8	30±6 kg/m^2^	Homens e mulheres hipertenso(a)s
Costa et al.^30^	n=19 mulheres hipertensas	0/100%	67,6±4,7	27,2 kg/m^2^	Mulheres ativas e inativas fisicamente
Boeno et al.^37^	n=13 homens pré-hipertensos	100%/0	22,7±2,6	25,3 kg/m^2^	Homens pré-hipertensos e inativos fisicamente
Maya et al.^36^	n=30 homens pré-hipertensos	100%/0	23±6,5	23,9 kg/m^2^	Homens pré-hipertensos e ativos fisicamente
Santos et al.^35^	n=15 hipertensos	NI	65,1±4,7	29,1 kg/m^2^	Homens e mulheres ativos fisicamente
Morales-Palomo et al.^34^	n=7 homens e mulheres com síndrome metabólica	57%/43%	55±9	29,1 kg/m^2^	Homens e mulheres com síndrome metabólica e normotensos
Morales-Palomo et al.^34^	n=7 homens	100%/0	59±6	33 kg/m^2^	Homens hipertensos com síndrome metabólica
Costa et al.^29^	n=14 homens	100%/0	24,9±4,1	24,2 kg/m^2^	Homens normotensos e ativos fisicamente
Graham et al.^33^	n=12 homens	100%/0	23±3	24 kg/m^2^	Homens pré-hipertensos e inativos fisicamente
Angadi et al.^27^	n=11 pré-hipertensos	91%/9%	24,6±3,7	24,4 kg/m^2^	Homens e mulheres pré-hipertensos
Lacombe et al.^32^	n=13 homens	100%/0	57±4	28,6 kg/m^2^	Homens pré-hipertensos e inativos fisicamente
Rossow et al.^26^	n=15 homens	100%/0	25,8±6,5	22,6 kg/m^2^	Homens normotensos e treinados
Rossow et al.^26^	n=10 mulheres	0/100%	25±3,4	22,2 kg/m^2^	Mulheres normotensas e treinadas
Mourot et al.^31^	n=10 homens	100%/0	24,6±0,6	21,86 kg/m^2^	Homens pré-hipertensos treinados

*Fonte: elaboração do Autor. Recife, 2019.*

**Tabela 2:** Características das sessões de EC e EI dos estudos incluídos

Autores	Modalidade	Local de intervenção/ Supervisão	Protocolo EI	Protocolo EC	Equipamento e momento de análise	Mecanismos relacionados a HPE
Pimenta et al.^38^	Esteira	Laboratório/Sim	5 x 3 min – 85-95% VO_2_res/ 2 min – 50-60% VO_2_res	~35 min – 60 - 70% VO_2_res	Esfigmomanômetro aneroide - 60min	Não investigado
Costa et al.^30^	Esteira	Laboratório/Sim	10 x 1 min – 80-85%FCres/ 2 min – 40-45%FCres	30 min – 50-55%FCres	Oscilométrico - 60min	**EI:** → DC, ↓ RVP, IVT, → CA; **EC:** → DC, → RVP, ↓ IVT, → CA
Boeno et al.^37^	Esteira	Laboratório/Sim	5 km: 1 min- 90% FCmáx/ 1 min -60% FCmáx	5 km – 70% FCmáx	Esfigmomanômetro digital - 60min	Não investigado
Maya et al.^36^	Esteira	Laboratório/Sim	500 kcal: 3 min – 115%LA/ 1min 30s RP	500 kcal: 85% LA	Esfigmomanômetro aneroide - 60min	Não investigado
Santos et al.^35^	Cicloergômetro	Laboratório/Sim	4x 4 min-85-90%FCres/ 2 min - 50%FCres	40 min - 60-80% FCres	Esfigmomanômetro aneroide - 60min	Não investigado
Morales-Palomo et al.^34^	Cicloergômetro	Laboratório/Sim	5 x 4 min-90% FCpico/ 3min 70% FCpico (~460 kcal)	~70 min-60% FCpico (~460 kcal)	Esfigmomanômetro digital – 45min	**EI:** ↑ DC, ↓ VS, ↓ RVP; **EC:** → DC, → VS, → RVP
Morales-Palomo et al.^34^	Cicloergômetro	Laboratório/Sim	5 x 4 min-90% FCpico/ 3 min 70% FCpico (~460 kcal)	~70 min-60% FCpico (~460 kcal)	Esfigmomanômetro digital – 45min	**EI:** ↑ DC, ↓ VS, ↓ RVP; **EC:** → DC, → VS, → RVP
Costa et al.^29^	Esteira	Laboratório/Sim	10 x 1 min-90% MAV/ 1min - 30% MAV	20 min - 60% MAV	Esfigmomanômetro digital – 60min	Não investigado
Graham et al.^33^	Cicloergômetro	Laboratório/Sim	5 x 30s - 0,075% MC - *all out* /4 min 30 s - RA – ergômetro de MMSS	50 min-65% VO_2_máx	Esfigmomanômetro aneroide - 60min	Não investigado
Graham et al.^33^	Cicloergômetro	Laboratório/Sim	5 x 30s - 0,075% MC - *all out* /4 min 30 s - RA – ergômetro de MMII	50 min-65% VO_2_máx	Esfigmomanômetro aneroide - 60min	Não investigado
Angadi et al.^27^	Cicloergômetro	Laboratório/Sim	4 x 4min-90-95%FCmáx/3min –50%FCmáx	30 min - 75-80% FCmáx	Oscilométrico - 60min	Não investigado
Angadi et al.^27^	Cicloergômetro	Laboratório/Sim	6 x 30s- (0,075% MC – *all out* ) /4min – 50%FCmáx	30 min - 75-80% FCmáx	Oscilométrico - 60min	Não investigado
Lacombe et al.^32^	Cicloergômetro	Laboratório/Sim	5x 2min - 85%VO_2_máx/ 2min-40%VO_2_máx	21 min - 60% VO_2_máx	Esfigmomanômetro digital - 60min	**EI:** ↓SBR, → DC, ↓VS. **EC:** → SBR, → DC, ↓VS
Rossow et al.^26^	Cicloergômetro	Laboratório/Sim	4 x 30s -0,07% MC – *all out* /4min30s- RA	60 min-60% FCres	Esfigmomanômetro digital - 60min	**EI:** ↑ DC, ↓ RVP; **EC:** ↑ DC, ↓ RVP
Mourot et al.^31^	Cicloergômetro	Laboratório/Sim	9x4min-1ºLV/ 1min-Ppico	48 min-1º LV	Esfigmomanômetro digital – 60min	Não investigado

*N: número de participantes; EI: exercício intervalado; EC: exercício contínuo; IMC: índice de massa corporal; LA: limiar anaeróbio; LV: limiar ventilatório; FCres: frequência cardíaca de reserva; FCmáx: frequência cardíaca máxima; Wmáx: Watts máximos; FCpico: frequência cardíaca de pico; Ppico: potência de pico; MAV: máxima velocidade aeróbia na esteira; VO_2_máx: consumo máximo de oxigênio; VO_2_res: consumo de oxigênio de reserva; MC: massa corporal; H: homens; M: mulheres; MMSS: membro superior; MMII: membro inferior; RA: recuperação ativa; RP: recuperação passiva; NI: não informado; DC: débito cardíaco; RVP: resistência vascular periférica; VS: volume sistólico; SBR: sensibilidade barorreflexa; IVT: impedância vascular total; CA: complacência arterial; ↑ aumento; ↓ redução; → manutenção. Fonte: elaboração do Autor. Recife, 2019.*

### Características das intervenções

Dos 12 estudos incluídos, sete (~58%) utilizaram cicloergômetro,^26 , 27 , 31 - 35^ e cinco utilizaram esteira^29 , 30 , 36 - 38^ nas sessões. Quando a sessão de EI foi realizada na esteira, foram observadas reduções sobre PA sistólica e diastólica de ~9,8 e 4,4 mmHg, respectivamente. Quando a sessão de EI foi realizada em cicloergômetro, a redução da PA sistólica e diastólica foi de ~7,6 e 3,7 mmHg, respectivamente. A redução da PA sistólica e diastólica após a sessão de EC na esteira foi de ~6,2 e 2,5 mmHg, respectivamente, e no cicloergômetro a redução da PA sistólica e diastólica foi de ~4,5 e 2,6 mmHg, respectivamente. O protocolo de EI mais utilizado consistiu em 4 minutos em alta intensidade, seguidos de 3 minutos,^27 , 34^ 2 minutos^35^ ou 1 minuto^31^ de recuperação ativa. Os outros protocolos utilizaram períodos mais curtos (30 segundos a 3 minutos) em alta intensidade. Já os protocolos de EC tiveram estímulo constante, com duração de 30 a 70 minutos.

A Tabela 3 mostra a avaliação qualitativa dos estudos incluídos. De acordo com a escala TESTEX (0 a 15 pontos), todos os estudos apresentaram pontuação acima de 10 pontos. Os pontos mais frágeis nos estudos foram: ausência de ocultação na alocação (92%),^26 - 29 , 31 - 37^ cegamento do avaliador para avaliação do desfecho (100%)^26 , 27 , 29 - 38^ e ausência de reporte sobre eventos adversos (75%).^26 , 29 - 31 , 33 - 37^

**Tabela 3:** Análise da qualidade metodológica dos estudos incluídos

Autores	Qualidade do estudo					Parcial (0 a 5)	Qualidade do estudo										Parcial (0 a 10)	Total (0 a 15)
	
	1	2	3	4	5		6 a	6 b	6 c	7	8 a	8 b	9	10	11	12		
Costa et al. (2020)	1	1	1	1	0	4	1	0*	-	1	1	1	1	NC	1	1	7	11
Pimenta et al. (2019)	1	1	0	1	0	3	1	1	-	1	1	1	1	NC	1	1	8	11
Boeno et al. (2019)	1	1	0	1	0	3	1	0*	-	1	1	1	1	NC	1	1	7	10
Maya et al. (2018)	1	1	0	1	0	3	1	0*	-	1	1	1	1	NC	1	1	7	10
Santos et al. (2018)	1	1	0	1	0	3	1	0*	-	1	1	1	1	NC	1	1	7	10
Morales-Palomo et al. (2017)	1	1	0	1	0	3	1	0*	-	1	1	1	1	NC	1	1	7	10
Costa et al. (2016)	1	1	0	1	0	3	1	0*	-	1	1	1	1	NC	1	1	7	10
Graham et al. (2016)	1	1	0	1	0	3	1	0*	-	1	1	1	1	NC	1	1	7	10
Angadi et al. (2015)	1	1	0	1	0	3	1	1	-	1	1	1	1	NC	1	1	8	11
Lacombe et al. (2011)	1	1	0	1	0	3	1	1	-	1	1	1	1	NC	1	1	8	11
Rossow et al. (2010)	1	1	0	1	0	3	1	0*	-	1	1	1	1	NC	1	1	7	10
Mourot et al. (2004)	1	1	0	1	0	3	1	0*	-	1	1	1	1	NC	1	1	7	10

* : estudos que não reportaram o número de desistências, porém todos finalizaram com o mesmo número de participantes que iniciaram a intervenção; 6c: não se enquadra, todos os estudos são de análise aguda; NC: sem grupo-controle. Qualidade dos estudos: 1 = critério de elegibilidade específico; 2 = tipo de randomização especificada; 3 = alocação ocultada; 4 = grupos similares no *baseline* ; 5 = os avaliadores foram cegados (pelo menos em um resultado principal); 6 = resultados avaliados em 85% dos participantes (6a = 1 ponto se concluíram mais de 85%; 6b = 1 ponto se os eventos adversos foram relatados; 6c = se for relatado atendimento ao exercício); 7 = intenção de tratar a análise estatística; 8 = comparação estatística entre os grupos foi relatada (8a = 1 ponto se comparações entre grupos são relatadas para a variável de desfecho primário de interesse; 8b = 1 ponto se comparações estatísticas entre grupos são relatadas para pelo menos uma medida secundária); 9 = medidas pontuais e medidas de variabilidade para todas as medidas de resultado que foram relatadas; 10 = monitoramento da atividade no grupo-controle; 11 = a intensidade relativa ao exercício permaneceu constante; 12 = o volume do exercício e o gasto de energia foram relatados. *Fonte* : elaboração do Autor. Recife, 2019.

### Efeito do EI versus EC sobre a pressão arterial clínica

**Figura 2:**
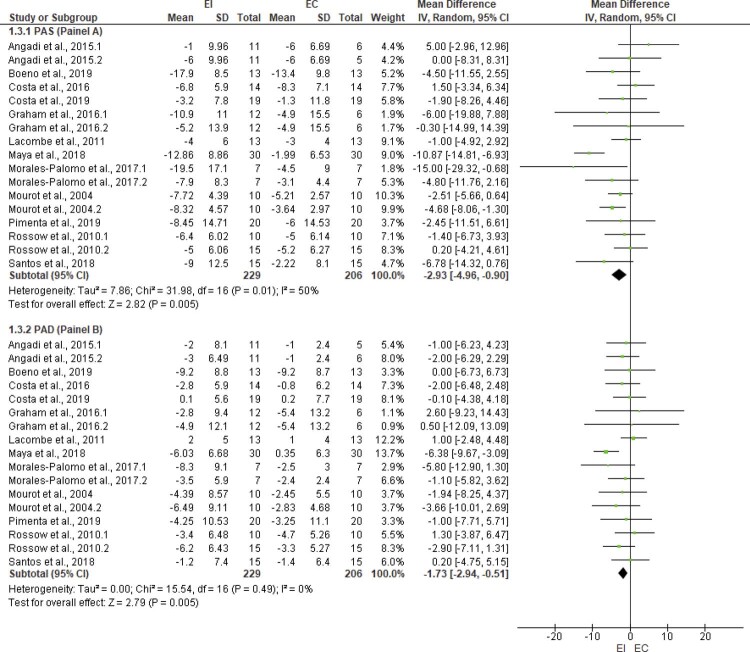
Forest plot da comparação dos efeitos do exercício intervalado (EI) versus exercício contínuo (EC) sobre a pressão arterial (PA) sistólica (painel A) e diastólica (painel B). Resultados expressos em delta de mudança (valores de pressão arterial pós-exercício – valores de pressão arterial pré-exercício).

A Figura 2 (painel A) mostra uma comparação direta entre os efeitos do EI e do EC sobre a PA sistólica. A metanálise demonstrou diferença significativa em favor do EI (WMD: -2,93 mmHg [IC 95%: -4,96, -0,90], p = 0,005). Observou-se moderada heterogeneidade para esta análise (I^2^= 50%; p = 0,01). Uma análise de sensibilidade mostrou que o efeito em favor do EI sobre a HPE permaneceu após a remoção de cada um dos estudos incluídos.

A comparação direta entre os efeitos do EI e do EC sobre a PA diastólica demonstrou diferença significativa em favor do EI (WMD: -1,73 mmHg [IC95%: -2,94, -0,51], p = 0,005). Baixa heterogeneidade foi encontrada para esta análise (I^2^= 0%; p = 0,49), conforme apresenta a Figura 2 (painel B). Na análise de sensibilidade, todos os estudos (um por um) foram retirados, e verificou-se que apenas a remoção do estudo de Maya et al.^36^ da análise faz com que o resultado positivo em favor do EI desaparecesse (WMD: -0,99 mmHg [IC95%: -2,30, 0,32], p = 0,14; I^2^= 0%; p = 0,97).

## Discussão

Pelo nosso conhecimento, essa é a primeira revisão sistemática e metanálise que comparou diretamente a magnitude da HPE após uma sessão de EC e EI em adultos. O principal achado desse estudo é que o EI apresenta redução da PA sistólica e diastólica de ~3 e 1,3 mmHg, respectivamente, maior que o EC (45 a 60 minutos pós-exercício). Entretanto, é importante destacar que esse resultado sobre a PA diastólica representa considerável influência de um único estudo.^36^

De modo geral, o presente estudo observou que o EI reduziu ~8 e 4 mmHg a PA sistólica e diastólica, respectivamente, 45 a 60 minutos pós-exercício. Já a redução observada após o EC foi de ~5 e 2,6 mmHg para a PA sistólica e diastólica, respectivamente, no mesmo período pós-exercício analisado. Portanto, a comparação direta ( *head-to-head* ) dos efeitos dessas intervenções confirmou a superioridade do EI em comparação ao EC no que se refere a magnitude da HPE sistólica e diastólica entre 45 e 60 minutos. Esses dados são similares aos encontrados em metanálise anterior,^11^ que observou redução da PA sistólica de 7,1 e 4,0 mmHg e redução da PA diastólica de 2,5 e 3,2 mmHg, respectivamente, para exercícios intervalados e contínuos. É importante destacar, porém, que não apenas a natureza intervalada *versus* contínua foi comparada na presente metanálise, e sim intervenções que envolveram especificamente EI (em intensidade vigorosa e “ *all out* ”) *versus* EC (em intensidade moderada e vigorosa), o que não foi feito no estudo anterior.^11^

Estudos têm demonstrado que a magnitude da HPE pode estar relacionada tanto com a intensidade atingida durante a sessão de exercício físico^10 , 11 , 39^ quanto com o volume do exercício.^11 , 40^ Na presente metanálise, a maioria dos estudos incluídos (~66%; n = 8)^29 - 32 , 34 , 36 - 38^ equalizou o volume, e/ou intensidade média, e/ou gasto energético total das sessões de EI com EC, o que pode facilitar o entendimento do impacto da natureza (intervalado *versus* contínua) e da intensidade dos estímulos sobre a magnitude da HPE. Tal aspecto é importante porque estudos mostram que, quando o volume e/ou a intensidade média são equalizados, a HPE é semelhante entre o EI e o EC.^41 , 42^ Contudo, dos estudos incluídos nessa revisão sistemática, naqueles que apresentaram volume, e/ou intensidade média, e/ou gasto energético total equalizados entre os protocolos de exercício, foram observadas reduções médias de -9,7 e -5 mmHg na PA sistólica e -4,3 e -2,2 mmHg na PA diastólica, para o EI e o EC, respectivamente. Os protocolos de EI que apresentaram menor volume, e/ou intensidade média, e/ou gasto energético,^26 , 27 , 33 , 35^ mostraram reduções médias de -6,2 e -3,4 mmHg na PA sistólica e diastólica, respectivamente, o que foi ligeiramente maior que as reduções médias de PA sistólica e diastólica observadas no EC (-4,9 e -3,2 mmHg, respectivamente). Portanto, os estímulos em alta intensidade parecem ter um papel na magnitude da HPE, independentemente de haver ou não equalização do volume e/ou da intensidade média e/ou do gasto energético total.

Os mecanismos pelos quais a HPE ocorre após a realização de uma sessão de EC são bem documentados.^13 , 16 , 43 , 44^ A redução da resistência vascular periférica tem sido frequentemente atribuída a um dos principais mecanismos de redução aguda da PA pós-exercício,^45^ que é auxiliada pela redução da atividade simpática no vaso, devido ao controle barorreflexo, o que gera vasodilatação prolongada.^46 , 47^ Além disso, vasodilatadores locais (p. ex., prostaglandinas e óxido nítrico) também desempenham papel importante para a ocorrência da HPE.^48 , 49^ Em pacientes com disfunções vasculares (p. ex., idosos, portadores de doença arterial periférica e obesos), a HPE ocorre por redução do volume sistólico, devido a diminuição da pré-carga, que não é compensada por aumento da frequência cardíaca.^26 , 45 , 50^ Os estudos que compararam diretamente os efeitos agudos do EC e do EI sobre a PA mostraram que os mecanismos relacionados à HPE entre esses modelos de exercício parecem ser diferentes.^26 , 30 , 32 , 34^

Em normotensos, Rossow et al.^26^ observaram maior redução da resistência vascular periférica e aumento do débito cardíaco (mediado por aumento da frequência cardíaca) após o protocolo de EI comparado ao EC. Em homens pré-hipertensos, Lacombe et al.^32^ demonstraram que o EI gerou maiores mudanças na sensibilidade barorreflexa e variabilidade da frequência cardíaca do que o EC no período pós-exercício. Morales-Palomo et al.^34^ observaram, em indivíduos com síndrome metabólica (normotensos e hipertensos), maiores reduções no volume sistólico, resistência vascular periférica, resistência vascular cutânea, maior fluxo sanguíneo na pele e maiores aumentos da frequência cardíaca após EI, em comparação a EC. Em mulheres hipertensas de meia-idade e idosas, Costa et al.^30^ observaram que 60 minutos após EI houve redução da resistência vascular periférica, em comparação à sessão-controle, o que não ocorreu após EC. Em conjunto, o EI parecer induzir maior redução da resistência vascular periférica pós-exercício, em comparação ao EC. É importante destacar que os estudos que compararam os determinantes hemodinâmicos da HPE entre EI e EC são poucos e envolvem diferentes populações, o que dificulta o entendimento das possíveis diferenças entre esses protocolos.

Do ponto de vista clínico, redução crônica de 2 mmHg na PA sistólica reduz em 6% o risco de mortalidade por acidente vascular cerebral e em 4% o risco de doença arterial coronariana, ao passo que redução de 5 mmHg diminui o risco em 14% e 9%, respectivamente.^15^ Uma metanálise demonstrou que o efeito anti-hipertensivo crônico do EI e do EC é similar em indivíduos com pré-hipertensão e hipertensão, tanto sobre a PA sistólica (-6,3 *versus* -5,8 mmHg) quanto sobre a diastólica (-3,8 *versus* -3,5 mmHg) em repouso.^19^ Em relação ao efeito anti-hipertensivo agudo do exercício, a presente revisão sugere superioridade do EI em relação ao EC tanto para a PA sistólica (~ 3 mmHg) quanto para a diastólica (~1,3 mmHg). Entretanto, é importante ressaltar que esse efeito foi observado entre 45 e 60 minutos pós-exercício. Portanto, exercício físico deve ser realizado com regularidade para que os benefícios crônicos sejam alcançados.

Os achados deste estudo demonstraram que uma única sessão de exercício aeróbio é capaz de promover HPE em adultos, independentemente do estímulo realizado (EC ou EI). A magnitude da HPE foi relacionada a intensidade e natureza intervalada do exercício, de tal forma que o EI gerou maior HPE. No entanto, é importante destacar que existem diferentes formas de prescrição de EI, o que impossibilita a determinação de um protocolo que maximize a HPE.

Apesar dos resultados interessantes e novos, essa revisão sistemática apresenta algumas limitações: i) apenas quatro bases de dados foram pesquisadas para inclusão dos estudos; ii) poucos estudos foram incluídos nessa revisão; iii) os estudos incluídos envolveram uma pequena quantidade de participantes (10 a 30 indivíduos); iv) diferentes métodos de aferição da PA foram utilizados nos estudos; v) o controle da ingestão alimentar e de água, o nível de atividade física e outros fatores de confusão foram pouco reportados nos estudos; vi) curto tempo de monitoração da PA pós-exercício, o que dificulta o entendimento da duração da HPE entre os protocolos.

## Conclusões

Essa revisão sistemática e metanálise de estudos cruzados sugere que, comparado ao EC, o EI induz uma HPE de maior magnitude entre 45 e 60 minutos pós-exercício em adultos, tanto na PA sistólica (~3 mmHg) quanto na diastólica (~1,3 mmHg). No entanto, a importância clínica desses achados deve ser considerada com cautela. São necessários mais estudos que comparem o efeito agudo do EI e do EC sobre a PA ambulatorial, a fim de esclarecer se de fato a diferença entre esses tipos de exercícios tem importância clínica no que se refere ao controle agudo da PA, tanto na vigília quanto no sono.
