# Supplementary material for: Efeito Agudo do Exercício Intervalado versus Contínuo sobre a Pressão Arterial: Revisão Sistemática e Metanálise
Source: Arq Bras Cardiol. 2020 Jul 28;115(1):5–14. [Article in Portuguese] doi: 10.36660/abc.20190107 (PMC8384323; doi:10.36660/abc.20190107)
Supplement: Translation

# Translation: Acute Effect of Interval vs. Continuous Exercise on Blood Pressure: Systematic Review and Meta-Analysis

**Authors:** Raphael José Perrier-Melo, Eduardo Caldas Costa, Breno Quintella Farah, Manoel da Cunha Costa

**Affiliations:** 1 Brazil Faculdade Pernambucana de Saúde, Recife, PE - Brazil; 2 Brazil Universidade Federal do Rio Grande do Norte - Educação Física, Natal, RN - Brazil; 3 Brazil Universidade Federal Rural de Pernambuco, Recife, PE - Brazil; 4 Brazil Universidade de Pernambuco - Educação Física, Recife, PE – Brazil

**Keywords:** Hypertension, Blood Pressure, Post-Exercise Hypotension, Exercise Therapy, Exercise, Review

## Abstract

**Background:**

Continuous aerobic exercise (CE) is one of the main non-pharmacological recommendations for hypertension prevention and treatment. CE is safe and effective to reduce blood pressure chronically, as well as in the first few hours after its performance, a phenomenon known as post-exercise hypotension (PEH). Interval exercise (IE) also results in PEH.

**Objective:**

This systematic review and meta-analysis sought to compare the magnitude of PEH between CE and IE in adults.

**Methods:**

A systematic review of studies published in journals indexed in the PubMed, Web of Knowledge, Scopus and CENTRAL databases was performed until March 2020, which compared the magnitude of PEH between CE and IE. PEH was defined as between 45-60 minutes post-exercise. The differences between groups on blood pressure were analyzed using the random effects model. Data were reported as weighted mean difference (WMD) and 95% confidence interval (CI). A p-value <0.05 was considered statistically significant. The TESTEX scale (0-15) was used to verify the methodological quality of the studies.

**Results:**

The IE showed a higher magnitude of PEH on systolic blood pressure (WMD: -2.93 mmHg [95% CI: -4.96, -0.90], p = 0.005, I2 = 50%) and diastolic blood pressure (WMD: -1,73 mmHg [IC95%: 2,94, -0,51], p= 0.005, I^2^= 0%) when compared to CE (12 studies, 196 participants). The scores of the studies on the TEXTEX scale varied from 10 to 11 points.

**Conclusions:**

The IE resulted in a higher magnitude of PEH when compared to CE between 45 and 60 minutes post-exercise. The absence of adverse event data during IE and CE in the studies prevents comparisons of the safety of these strategies. (Arq Bras Cardiol. 2020; 115(1):5-14)

## Introduction

Hypertension affects between 30 and 40% of the world’s population.^1 , 2^ In Brazil, its prevalence varies from 22.3 to 43.9%, affecting more than 60% of the elderly.^3 , 4^ Hypertension is directly associated with the incidence of heart and cerebrovascular diseases,^3^ responsible for approximately 20% of deaths in individuals over 30 years of age,^5^ in addition to generating costs of around R$ 30.8 billion *reais* per year.^6^ Changes in lifestyle, including physical activity, healthy eating habits, weight reduction and smoking cessation have been strongly recommended for the prevention and treatment of hypertension.^1 , 3^ In fact, changes in lifestyle result in reductions in blood pressure (BP) levels, which reduce the risk of cardiovascular events.^3 , 7 , 8^

Regarding physical exercises, the guidelines for the prevention and treatment of hypertension recommend aerobic exercises performed continuously (CE), mainly of moderate intensity, as they are safe and effective for reducing BP levels, improving the cardiovascular and metabolic risk profile, in addition to increasing cardiorespiratory fitness.^3 , 9^ The antihypertensive effects of CE can occur acutely,^10 , 11^ a phenomenon known as post-exercise hypotension (PEH), or chronically, after several sessions of physical exercise over weeks or months.^12 , 13^ In recent years, special attention has been given to exercises that can enhance the magnitude and duration of PEH, considering that this effect would reduce cardiovascular overload in the hours after the exercise session, thus decreasing the risk of cardiovascular events.^14 , 15^ Additionally, more recent studies have shown that individuals with greater PEH after an exercise session, tend to have a greater reduction in resting BP after weeks of training (i.e., greater chronic effect).^16^ Therefore, the magnitude of PEH seems to predict the magnitude of the chronic antihypertensive effect, which represents an important practical applicability.

PEH can occur with different “doses” of physical exercise, both aerobic and strength.^16^ In relation to aerobic exercises, a systematic review and previous meta-analysis^11^ showed that PEH occurs after performing CE and IE, despite being documented mainly after CE, which is the basis for hypertension prevention and treatment recommendations.^3 , 9^ However, in recent years, IE, whether at vigorous or maximum intensity (“all out”), has been considered an alternative to CE for the improvement of several cardiovascular parameters, such as cardiorespiratory capacity,^17^ vascular function^18^ and clinical BP.^19^

However, it is important to highlight that no direct comparisons were made on the acute effects of CE and IE on BP. Thus, it is not clear whether there is a superiority of the acute antihypertensive effect between exercises, which is an important knowledge gap, as it can help professionals in both hypertension prevention and treatment. Therefore, the aim of this systematic review and meta-analysis was to compare the magnitude of PEH between CE and IE in adults.

## Methods

### Literature search strategy

The systematic review was carried out following the guidelines of the ‘Preferred Reporting Items for Systematic Reviews and Meta-analysis (PRISMA)’.^20^ The search for the articles was carried out in PubMed, Web of Knowledge, Scopus and CENTRAL electronic databases. The search strategy used the following descriptors and free terms: “high intensity interval training” [MeSH Terms] OR “high intensity interval exercise” [TIAB] OR “aerobic interval training” [TIAB] OR “aerobic interval exercise” [TIAB] OR “sprint training” [TIAB] OR “sprint” [TIAB] OR “sprint exercise” [TIAB] OR “sprint interval exercise” [TIAB] AND “blood pressure” [MeSH Terms] OR “post-exercise hypotension” [Mesh Terms] OR “post-exercise hypotension” [Mesh Terms] OR “hypotension” [Mesh Terms]. All processes for article search, selection and evaluation were carried out in duplicate and independently.

### Eligibility criteria

The eligibility criteria were established according to the PICOS (Population, Intervention, Comparator, Outcomes and Study Design) question.

### Population

This review included studies involving adults (18 years or older) of both genders, with no restriction regarding the level of physical activity and BP classification (normotensive, pre-hypertensive and hypertensive). Mean pre-exercise systolic and diastolic BP values were used to classify individuals regarding BP, following the same procedures as other systematic reviews^19 , 21^ and the 7^th^Brazilian Guidelines on Hypertension.^3^

### Intervention

The classification system for IE proposed by Weston et al.^22^ was used to define the eligibility criteria for this intervention. According to this proposal, repeated stimuli at vigorous intensity (80-100% of peak heart rate - HRpeak) interspersed with periods of recovery (active or passive) are classified as high-intensity interval training, and maximum stimuli (“all out”; or above the peak oxygen consumption load -VO_2_peak) interspersed with recovery periods (active or passive) are classified as sprint interval exercise. Studies that used the percentage of VO_2_peak, VO_2_reserve or rating of perceived exertion (RPE) equivalent to 80-100% of HRpeak according to the American College of Sports Medicine,^23^ were considered eligible, as well as the “all out” protocols. Studies that showed interventions associated with IE, such as another form of exercise (e.g., strength exercises) or nutritional strategy, were not considered for inclusion.

### Comparator

The CE was considered as a comparator of the IE. Studies that used the percentage of VO_2_peak, VO_2_reserve or RPE equivalent to moderate intensity (i.e., 64-76% of HRpeak) or vigorous intensity (i.e. 77-95% of HRpeak) were considered eligible. Studies that showed interventions associated with CE, such as another type of exercise or nutritional strategy, were not considered for inclusion.

### Outcomes

The primary outcome of this review was clinical BP, measured between 45 and 60 minutes post-exercise. This post-exercise time was defined considering that most studies that investigated the effects of CE and IE included measures within that period. Therefore, even though the study analyzed BP beyond 60 minutes post-exercise, this measure was not considered for the meta-analysis.

### Study Design

Crossover studies were considered, involving a session of CE and IE, randomized performance order, in English or Portuguese. The search was carried out without a date limit and ended in March 2020.

### Data extraction

An electronic spreadsheet was used to extract data from the included articles, according to the eligibility criteria, in duplicate and independently. In case of disagreement, a meeting was held, and a consensus was established between the researchers. The characteristics of the study participants (age, gender, body mass index, level of physical activity, BP classification), characteristics of the exercise sessions (modality, environments, duration, intensity and time spent in the training session), method of BP measurement and post-exercise BP measurement period were extracted and recorded. Absent data in the texts were requested directly from the authors.

### Evaluation of study methodological quality

The ‘Tool for the assEssment of Study qualiTy and reporting in Exercise (TESTEX)’ scale was used to assess the methodological quality of the included studies,^24^ also in duplicate and independently. In case of disagreement, a meeting was held, and consensus was established between the researchers.

### Quantitative synthesis

The changes (post and pre-intervention) in clinical BP were extracted from each study and expressed as mean ± standard deviation. The data were reported as weighted mean differences (WMD) and 95% confidence interval (95%CI). The heterogeneity (I^2^) between the studies was calculated. Values > 75% and p <0.10 were used to indicate high heterogeneity.^25^ The random-effects model was adopted in the presence of low or high statistical heterogeneity. Publication bias was assessed using the funnel plot ( Figure 3 ). The meta-analysis was performed using the Review Manager software (RevMan 5.3, Nordic Cochrane, Denmark). Two studies did not report the standard deviation values in the pre- and post-intervention moments.^26 , 27^ In this case, the values were estimated based on the recommendations of Follman et al.^28^ For this purpose, the study by Costa et al.^29^ was adopted as the basis. In all analyses, the level of significance adopted was 5%.

**Figure 3:** Funnel plot of the comparison of interval exercise (IE) vs. continuous exercise (CE) on blood pressure (BP)

## Results

### Included studies

The search strategy identified 3,252 articles for the initial analysis. After screening the titles, abstracts and excluding duplicate results, 84 studies were selected for full-text analysis. Of these, 72 did not meet the eligibility criteria for inclusion in the study. Additionally, an unpublished study was included in the analyses.^30^Figure 1 shows the flowchart of the research results.

**Figure 1:** PRISMA flowchart of selected studies. BP: blood pressure; CE: aerobic exercises performed continuously; IE: interval exercise; PEH: post-exercise hypotension.

### Characteristics of participants

The 12 articles included in the study analyzed clinical BP as the main outcome and none of them reported adverse effects (n = 196; age between 20-75 years; BMI between 21.2-33.0 kg/m^2^).^26 , 27 , 29 - 38^ Of these, three studies involved 46 normotensive individuals (n = 23 women),^26 , 29 , 34^ with a mean age of 32.67 years, and mean BMI of 24.52 km/m^2^. The mean systolic and diastolic BP at rest was 118/65.46 mmHg in IE and 117.27 / 64.73 mmHg in CE. Six studies involved 89 pre-hypertensive patients (n = 1 woman),^27 , 31 - 33 , 36 , 37^ with a mean age of 29.15 years, and mean BMI of 24.68 km/m^2^. Mean systolic and diastolic BP at rest was 127.22 / 73.12 mmHg in IE and 126.72 / 73.22 mmHg in CE. Four studies involved 61 hypertensive patients (n = 34 women),^30 , 34 , 35 , 38^ mean age of 60,67 years, and mean BMI of 29,97 km/m^2^and all used antihypertensive medication.

Regarding the BP measurement, of the 12 included studies, four used the auscultatory method (~ 33%), while the others used the oscillometric method in an automatic equipment. All studies used inferential statistics, adopting a value of p ≤ 0.05. Table 1 and 2 shows additional information on the characteristics of the studies and interventions.

**Table 1:** Characteristics of participants included in the studies

Authors	Participants	Men (%) / Women (%)	Age (years)	BMI (kg/m^2^)	Sample characteristic
Pimenta et al.^38^	n=20 (15 women)	25%/75%	51±8 years	30±6 kg/m^2^	Hypertensive men and women
Costa et al.^30^	n= 19 hypertensive women	0/100%	67.6±4.7 years	27.2 kg/m^2^	Physically active and inactive women
Boeno et al.^37^	n= 13 pre-hypertensive men	100%/0	22.7±2.6 years	25.3 kg/m^2^	Pre-hypertensive and physically inactive men
Maya et al.^36^	n= 30 pre-hypertensive men	100%/0	23±6.5 years	23.9 kg/m^2^	Pre-hypertensive and physically active men
Santos et al.^35^	n=15 hypertensive	NI	65.1±4.7 years	29.1 kg/m^2^	Physically active men and women
Morales-Palomo et al.^34^	n=7 men and women with metabolic syndrome	57%/43%	55±9 years	29.1 kg/m^2^	Normotensive men and women with metabolic syndrome
Morales-Palomo et al.^34^	n= 7 men	100%/0	59±6 years	33 kg/m^2^	Hypertensive men with metabolic syndrome
Costa et al.^29^	n= 14 men	100%/0	24.9±4.1 years	24.2 kg/m^2^	Normotensive and physically active men
Graham et al.^33^	n=12 men	100%/0	23±3 anos	24 kg/m^2^	Pre-hypertensive and physically inactive men
Angadi et al.^27^	n=11 pre-hypertensive individuals	91%/9%	24.6±3.7 years	24.4 kg/m^2^	Pre-hypertensive men and women
Lacombe et al.^32^	n=13 men	100%/0	57±4 years	28.6 kg/m^2^	Pre-hypertensive and physically inactive men
Rossow et al.^26^	n= 15 men	100%/0	25.8±6.5 years	22.6 kg/m^2^	Trained normotensive men
Rossow et al.^26^	n=10 women	0/100%	25±3.4 years	22.2 kg/m^2^	Trained normotensive women
Mourot et al.^31^	n=10 men	100%/0	24.6±0.6 years	21.86 kg/m^2^	Trained pre-hypertensive men

*SOURCE: The author. Recife, 2019.*

**Table 2:** Characteristics of the CE and IE sessions of the included studies

Authors	Modality	Intervention site/ Supervision	IE Protocol	CE Protocol	Equipment and moment of analysis	Mechanisms related to PEH
Pimenta et al.^38^	Treadmill	Laboratory/Yes	5x 3 min – 85-95% resVO_2_/ 2 min – 50-60% resVO_2_	~35min – 60 - 70% res VO_2_	Aneroid sphygmomanometer - 60min	Not investigated
Costa et al.^30^	Treadmill	Laboratory/Yes	10x 1 min – 80-85% RHR/ 2min – 40-45% RHR	30 min – 50-55% RHR	Oscillometric - 60min	**IE:** → CO, ↓ PVR, ↓ TVI, → AC; **CE:** → CO, → PVR, ↓ TVI, → AC
Boeno et al.^37^	Treadmill	Laboratory/Yes	5 km: 1 min- 90% HRmax/ 1min -60% HRmax	5 km – 70% HRmax	Digital sphygmomanometer - 60min	Not investigated
Maya et al.^36^	Treadmill	Laboratory/Yes	500 kcal: 3 min – 115%AT/ 1min 30s PR	500 kcal: 85% AT	Aneroid sphygmomanometer - 60min	Not investigated
Santos et al.^35^	Cycle ergometer	Laboratory/Yes	4x 4 min-85-90% RHR/ 2min - 50% RHR	40 min - 60-80% RHR	Aneroid sphygmomanometer - 60min	Not investigated
Morales-Palomo et al.^34^	Cycle ergometer	Laboratory/Yes	5 x 4 min-90% HRpeak/ 3 min 70% HRpeak (~460 kcal)	~70 min-60% HRpeak (~460 kcal)	Digital sphygmomanometer – 45min	**IE:** ↑ CO, ↓ SV, ↓ PVR; **CE:** → CO, → SV, → PVR
Morales-Palomo et al.^34^	Cycle ergometer	Laboratory/Yes	5 x 4min-90% HRpeak/ 3 min 70% HRpeak (~460 kcal)	~70 min-60% HRpeak (~460 kcal)	Digital sphygmomanometer – 45min	**IE:** ↑ CO, ↓ SV, ↓ PVR; **CE:** → CO, → SV, → PVR
Costa et al.^29^	Treadmill	Laboratory/Yes	10x 1 min-90% MAV/ 1 min - 30% MAV	20 min - 60% MAV	Digital sphygmomanometer – 60min	Not investigated
Graham et al.^33^	Cycle ergometer	Laboratory/Yes	5x 30 s – 0.075% BM - all out/4 min 30 s - AR – UULL ergometer	50 min-65% VO_2_max	Aneroid sphygmomanometer - 60min	Not investigated
Graham et al.^33^	Cycle ergometer	Laboratory/Yes	5x 30s – 0.075% BM - all out/4 min 30 s - AR – LLLL ergometer	50 min-65% VO_2_max	Aneroid sphygmomanometer - 60min	Not investigated
Angadi et al.^27^	Cycle ergometer	Laboratory/Yes	4 x 4min-90-95% HRmax/3 min –50% HRmax	30 min - 75-80% HRmax	Oscillometric - 60min	Not investigated
Angadi et al.^27^	Cycle ergometer	Laboratory/Yes	6 x 30s- (0.075% BM – all out) /4min – 50% HRmax	30 min - 75-80%HRmax	Oscillometric - 60min	Not investigated
Lacombe et al.^32^	Cycle ergometer	Laboratory/Yes	5x 2min - 85% VO_2_max/ 2min-40% VO_2_max	21 min - 60% VO_2_max	Digital sphygmomanometer - 60min	**IE:** ↓BRS, → CO, ↓SV. **CE:** BRS, → CO, ↓SV
Rossow et al.^26^	Cycle ergometer	Laboratory/Yes	4x 30 s -0.07% BM – all out /4 min 30 s- AR	60 min-60% RHR	Digital sphygmomanometer - 60min	**IE:** ↑ CO, ↓ PVR; **CE:** CO, ↓ PVR
Mourot et al.^31^	Cycle ergometer	Laboratory/Yes	9x4 min-1^st^VT/ 1 min-Ppeak	48 min-1^st^VT	Digital sphygmomanometer – 60min	Not investigated

*N: number of participants; IE: interval exercise; CE: continuous exercise; BMI: body mass index; AT: anaerobic threshold; VT: ventilatory threshold; RHR: reserve heart rate; HRmax: maximum heart rate; Wmax: maximum Watts; HRpeak: peak heart rate; Ppeak: peak power; MAV: maximum aerobic velocity on the treadmill; VO_2_max: maximum oxygen consumption; VO_2_res: reserve oxygen consumption; BM: body mass; M: men; W: women; UULL: upper limb; LLLL: lower limb; AR: active recovery; PR: passive recovery; NI: not informed; CO: cardiac output; PVR: peripheral vascular resistance; SV: stroke volume; BRS: baroreflex sensitivity; TVI: total vascular impedance; AC: arterial compliance; ↑ increase; ↓ reduction; → maintenance. SOURCE: The author. Recife, 2019.*

### Characteristics of interventions

Of the 12 studies included, seven (~ 58%) used a cycle ergometer,^26 , 27 , 31 - 35^ and five used a treadmill^29 , 30 , 36 - 38^ in the exercise sessions. When the IE session was performed on the treadmill, reductions in systolic and diastolic BP of ~ 9.8 and 4.4 mmHg were observed, respectively. When the IE session was performed on a cycle ergometer, the reduction in systolic and diastolic BP was ~ 7.6 and 3.7 mmHg, respectively. The reduction in systolic and diastolic BP after the CE session on the treadmill was ~ 6.2 and 2.5 mmHg, respectively, and the reduction in systolic and diastolic BP in the cycle ergometer was ~ 4.5 and 2.6 mmHg, respectively. The most frequently used IE protocol consisted of 4 minutes at high intensity, followed by 3 minutes,^27 , 34^ 2 minutes^35^ or 1 minute^31^ of active recovery. The other protocols used shorter periods (30 seconds to 3 minutes) at high intensity. The CE protocols, on the other hand, had a constant stimulus, lasting between 30 and 70 minutes.

Table 3 shows the qualitative assessment of the included studies. According to the TESTEX scale (0-15 points), all studies had scores > 10 points. The weakest points in the studies were: lack of allocation concealment (92%),^26 - 29 , 31 - 37^ blinding of the evaluator to evaluate the outcome (100%)^26 , 27 , 29^ and absence of the reporting of adverse events (75%).^26 , 29 - 31 , 33 - 37^

**Table 3:** Methodological quality analysis of the included studies

Authors	Study quality					Partial (0-5)	Study quality										Partial (0-10)	Total (0-15)
	
	1	2	3	4	5		6 a	6 b	6 c	7	8 a	8 b	9	10	11	12		
Costa et al. 2020	1	1	1	1	0	4	1	0*	-	1	1	1	1	NC	1	1	7	11
**Pimenta et al. 2019**	**1**	**1**	**0**	**1**	**0**	**3**	**1**	**1**	-	**1**	**1**	**1**	**1**	**NC**	**1**	**1**	**8**	**11**
Boeno et al. 2019	1	1	0	1	0	3	1	0*	-	1	1	1	1	NC	1	1	7	10
Maya et al. 2018	1	1	0	1	0	3	1	0*	-	1	1	1	1	NC	1	1	7	10
Santos et al. 2018	1	1	0	1	0	3	1	0*	-	1	1	1	1	NC	1	1	7	10
Morales-Palomo et al. 2017	1	1	0	1	0	3	1	0*	-	1	1	1	1	NC	1	1	7	10
Costa et al. 2016	1	1	0	1	0	3	1	0*	-	1	1	1	1	NC	1	1	7	10
Graham et al. 2016	1	1	0	1	0	3	1	0*	-	1	1	1	1	NC	1	1	7	10
Angadi et al. 2015	1	1	0	1	0	3	1	1	-	1	1	1	1	NC	1	1	8	11
Lacombe et al. 2011	1	1	0	1	0	3	1	1	-	1	1	1	1	NC	1	1	8	11
Rossow et al. 2010	1	1	0	1	0	3	1	0*	-	1	1	1	1	NC	1	1	7	10
Mourot et al. 2004	1	1	0	1	0	3	1	0*	-	1	1	1	1	NC	1	1	7	10

*- studies that did not report the number of dropouts; however, all ended with the same number of participants who started the intervention, 6c- does not fit, all studies show an acute analysis, NC - no control group. Quality of studies: 1 = Specific eligibility criterion; 2 = Type of randomization specified; 3 = Allocation concealment; 4 = Similar groups in the baseline; 5 = The evaluators were blinded (at least for one main result); 6 = Results evaluated in 85% of participants (6a = 1 point if more than 85% were concluded; 6b = 1 point if adverse events were reported; 6c = if exercise attendance is reported); 7 = Intention to treat statistical analysis; 8 = Statistical comparison between groups were reported (8a = 1 point if comparisons between groups are reported for the variable primary outcome of interest; 8b = 1 point if statistical comparisons between groups are reported for at least one secondary measure); 9 = Point measures and measures of variability for all outcome measures were reported; 10 = Monitoring of activity in the control group; 11 = The intensity related to the exercise remained constant; 12 = Exercise volume and energy expenditure were reported. SOURCE: The author. Recife, 2019.

### Effect of IE versus CE on clinical BP

Figure 2 (panel A) shows the direct comparison between the effects of IE and CE on systolic BP. The meta-analysis showed a significant difference in favor of IE (WMD: -2.93 mmHg [95% CI: -4.96, -0.90], p = 0.005). Moderate heterogeneity was found for this analysis (I^2^= 50%; p = 0.01).

**Figure 2:** Forest plot of the comparison of the effects of interval exercise (IE) vs. continuous exercise (CE) on systolic (panel A) and diastolic (panel B) blood pressure (BP). Results are expressed in delta change (post-exercise blood pressure values - pre-exercise blood pressure values).

A sensitivity analysis showed that the effect in favor of IE on PEH persisted after the removal of each of the included studies.

The direct comparison between the effects of IE and CE on diastolic BP showed a significant difference in favor of IE (WMD: -1.73 mmHg [95% CI: -2.94, -0.51], p = 0.005). Low heterogeneity was found for this analysis (I^2^= 0%; p = 0.49), as shown in Figure 2 (panel B). In the sensitivity analysis, all studies (one by one) were removed and it was found that only the removal of the study by Maya et al.^36^ from the analysis made the positive result in favor of IE disappear (WMD: -0.99 mmHg [95% CI: -2.30, 0.32], p = 0.14; I^2^= 0%; p = 0.97).

## Discussion

To the best of our knowledge, this is the first systematic review and meta-analysis that directly compared the magnitude of PEH after a session of CE and IE in adults. The main finding of this study is that the IE shows a reduction in systolic and diastolic BP of ~ 3 and 1.3 mmHg, respectively, more than the CE (between 45-60 minutes post-exercise). However, it is important to highlight that this result on diastolic BP has considerable influence of a single study.^36^

Overall, the present study observed that IE showed a reduction of ~ 8 and 4 mmHg for systolic and diastolic BP, respectively, between 45-60 minutes post-exercise. The reduction observed after CE, however, was ~ 5 and 2.6 mmHg for systolic and diastolic BP, respectively, in the same post-exercise analyzed period. Therefore, a direct comparison (head-to-head) of the effects of these interventions confirmed the superiority of IE over CE in terms of the magnitude of systolic and diastolic PEH between 45-60 minutes. These data are similar to those found in a previous meta-analysis,^11^ which observed a reduction in systolic BP of 7.1 and 4.0 mmHg and a reduction in diastolic BP of 2.5 and 3.2 mmHg, respectively, for interval and continuous exercise. However, it is important to highlight that not only the interval *versus* continuous nature was compared in the present meta-analysis, but interventions that specifically involved IE (at vigorous intensity and “all out”) versus CE (at moderate and vigorous intensity), which was not performed in the previous study.^11^

Studies have shown that the magnitude of PEH can be related both to the intensity reached during the exercise session,^10 , 11 , 39^ and to the exercise volume.^11 , 40^ In the present meta-analysis, most of the included studies ( ~ 66%; n = 8)^29^ equalized the volume, and / or average intensity, and / or total energy expenditure of IE with CE sessions, which can facilitate the understanding of the impact of the exercise nature (interval *vs* . continuous) and intensity of stimuli on the PEH magnitude. This aspect is important because studies show that when volume and/or mean intensity are equalized, PEH is similar between IE and CE.^41 , 42^ However, of the studies included in this systematic review, those that showed volume, and / or mean intensity, and / or total energy expenditure equalized between the exercise protocols, mean reductions of -9.7 and -5 mmHg were observed in systolic BP and -4.3 and -2.2 mmHg in diastolic BP, for IE and CE, respectively. The IE protocols that showed lower volume, and/or mean intensity and/or energy expenditure,^26 , 27 , 33 , 35^ showed mean reductions of -6.2 and -3.4 mmHg in systolic and diastolic BP, respectively, which was slightly higher than the mean reductions in systolic and diastolic BP observed in CE (-4.9 and -3.2 mmHg, respectively). Therefore, high-intensity stimuli seem to have a role in the magnitude of PEH, regardless of whether or not there was volume, and/or mean intensity and / or total energy expenditure equalization.

The mechanisms through which PEH occurs after a CE session are well documented.^13 , 16 , 43 , 44^ The reduction in peripheral vascular resistance has often been attributed as one of the main mechanisms of acute post-exercise BP reduction,^45^ which is aided by the reduction of sympathetic activity in the vessel due to baroreflex control, which generates prolonged vasodilation.^46 , 47^ Additionally, local vasodilators, such as prostaglandins and nitric oxide, also play an important role in the occurrence of PEH.^48 , 49^ In patients with vascular disorders (e.g., the elderly, peripheral arterial disease, and obese individuals), PEH occurs by reducing the stroke volume, due to a decreased preload, which is not compensated by increased heart rate.^26 , 45 , 50^ The studies that directly compared the acute effects of CE and IE on BP showed that the mechanisms related to PEH between these exercise models seem to be different.^26 , 30 , 32 , 34^

In normotensive individuals, Rossow et al.^26^ observed a greater reduction in peripheral vascular resistance and an increase in cardiac output (mediated by an increase in heart rate) after the IE protocol, when compared to the CE. In pre-hypertensive men, Lacombe et al.^32^ demonstrated that IE resulted in greater changes in baroreflex sensitivity and heart rate variability than CE in the post-exercise period. Morales-Palomo et al.^34^ observed, in individuals with metabolic syndrome (normotensive and hypertensive), greater reductions in stroke volume, peripheral vascular resistance, skin vascular resistance, higher blood flow in the skin and greater increases in heart rate after IE, when compared to CE. In middle-aged and elderly hypertensive women, Costa et al.^30^ found that there was a reduction in peripheral vascular resistance 60 minutes after IE, when compared to the control session, which did not occur after CE. Considered together, IE seems to induce a greater reduction in peripheral vascular resistance post-exercise, when compared to CE. It is important to emphasize that the studies that compared the hemodynamic determinants of PEH between IE and CE are few and involve different populations, which makes it difficult to understand the possible differences between these protocols.

From a clinical point of view, a chronic reduction of 2 mmHg in systolic BP reduces the risk of mortality from stroke by 6% and coronary artery disease by 4%, while a reduction of 5 mmHg decreases 14% and 9% of the risk, respectively.^15^ A meta-analysis showed that the chronic antihypertensive effect of IE and CE is similar in individuals with prehypertension and hypertension, both on systolic (-6.3 *vs* . -5.8 mmHg) and diastolic BP (-3.8 *vs* . -3.5 mmHg) at rest.^19^ Regarding the acute antihypertensive effect of exercise, the present review suggests the superiority of IE over CE for both systolic (~ 3 mmHg) and diastolic (~ 1.3 mmHg) BP. However, it is important to note that this effect was observed between 45-60 minutes after the exercise. Therefore, physical exercise must be performed regularly so that the chronic benefits can be attained.

The findings of this study demonstrated that a single session of aerobic exercise is capable of promoting PEH in adults, regardless of the performed stimulus (CE or IE). The magnitude of the PEH was associated to the intensity and interval nature of the exercise, so that the IE generated a greater PEH. However, it is important to emphasize that there are different forms of IE prescription, which makes it impossible to determine a protocol that maximizes PEH.

Despite the new and interesting results, this systematic review has some limitations: i) only four databases were searched for study inclusion; ii) few studies were included in this review; iii) the included studies involved a small number of participants (between 10 and 30 individuals); iv) different BP measurement methods were used in the studies; v) food and water intake control, level of physical activity and other confounding factors were seldom reported in the studies; vi) short post-exercise BP monitoring time, which makes it difficult to understand the duration of PEH between protocols.

## Conclusions

This systematic review and meta-analysis of crossover studies suggests that IE induces a PEH of greater magnitude compared to CE, between 45-60 minutes post-exercise in adults, both in systolic (~3 mmHg) and diastolic BP (~1.3 mmHg). However, the clinical importance of these findings should be considered with caution. Future studies comparing the acute effect of IE and CE on ambulatorial BP are required in order to clarify whether, in fact, the difference between these types of exercises has clinical importance regarding acute BP control, both in wakefulness and in sleep.
